# Schwann Cell-Derived CCL2 Promotes the Perineural Invasion of Cervical Cancer

**DOI:** 10.3389/fonc.2020.00019

**Published:** 2020-01-29

**Authors:** Ting Huang, Qiong Fan, Yiwei Wang, Yunxia Cui, Zhihua Wang, Linlin Yang, Xiao Sun, Yudong Wang

**Affiliations:** ^1^Department of Gynecology, International Peace Maternity and Child Health Hospital, Shanghai Jiao Tong University School of Medicine, Shanghai, China; ^2^Shanghai Municipal Key Clinical Specialty, Shanghai, China; ^3^Shanghai Public Health Clinical Center, Female Tumor Reproductive Specialty, Shanghai, China; ^4^Shanghai Key Laboratory of Embryo Original Disease, Shanghai, China

**Keywords:** perineural invasion, Schwann cell, cervical cancer, CCL2/CCR2 axis, tumor microenvironment

## Abstract

Perineural invasion (PNI) has guiding significances for nerve preservation in cervical cancer, but there is no definite marker indicating PNI. Two cervical cancer cell lines (HeLa and ME-180) showed significant abilities to migrate along neurites *in vitro* and *in vivo*. Morphological observation revealed that Schwann cells (SC) arrived at the sites of cervical cancer cells before the onset of cancer metastasis. We used high-throughput antibody array to screen the signals mediating the interaction of nerve cells and cancer cells and found the high expression of CCL2 in dorsal root ganglion (DRG). Meanwhile, serum CCL2 showed a notable raise especially in cervical adenocarcinoma. SC-derived CCL2 bound to its receptor CCR2 and promoted the proliferation, migration, invasion, and epithelial-mesenchymal transition (EMT) of cervical cancer cells. In turn, cancer cell-derived signals triggered the expression of metalloproteinases (MMPs) including MMP2, MMP9, and MMP12 in SCs, promoting SCs to dissolve matrix. These data demonstrated that the cancer-nerve crosstalk formed a tumor microenvironment (TME) that facilitated to PNI. We identified the CCL2/CCR2 axis as a potential marker to predict the PNI and affect the nerve preservation for cervical cancer.

## Background

Cervical cancer is one of the most common gynecologic malignant tumors worldwide, accounting for ~13,000 of new cancer cases each year, and nearly 4,000 deaths (1). Despite the progress in surgical and conservative treatment options for cervical cancer, some patients still have pelvic cancer recurrence and distant metastasis. One of the factors affecting local recurrence or metastasis of cervical cancer is the occurrence of perineural invasion (PNI). PNI is the neoplastic invasion of nerves by cancer cells, a process that may prove to be another metastatic route (2). The occurrence of PNI was reported between 7 and 41.7% in previous studies investigating PNI in uterine cervical cancer (3–7). PNI is predicted to reduce the survival rate of cervical cancer patients (8–10). Thus, elucidating the mechanism of PNI and identifying markers for its early prediction are of great value for patients with early recurrence or metastasis of cervical cancer.

The tumor microenvironment (TME) is formed by cancer cells, stromal fibroblasts, altered extracellular matrix (ECM), vessels, and immune components. The peripheral nerve is a novel component of the TME (11, 12). The signals in the TME mediate the crosstalk between nerve cells and cancer cells, and lead to both cancer progression and nerve expansion, thus causing PNI (13). The abnormal release of chemotactic factors might be correlated with PNI in cancers (14, 15). Chemokine (C-C motif) ligand 2 (CCL2), also known as monocyte chemoattractant protein-1 (MCP-1), recruits monocytes and macrophages to the sites of inflammation (16). Previous studies have revealed that CCL2 secreted by the nerves facilitates pancreatic ductal adenocarcinoma invasion by binding to its receptor (CCR2) expressed on the membrane of cancer cells or macrophages (17, 18). Moreover, CCL2 was regarded as a molecular target of PNI in prostate cancer (18). CCL2 is related to both primary tumor development and metastasis in various cancers including cervical cancer (14, 19). Thus, the role of CCL2 and its receptor in PNI of cervical cancer is an emerging field of research.

Schwann cells (SCs) are a major component of the peripheral nerves and play an important role in promoting axon regeneration during repair (20). A recent study showed that SCs were highly cancer-affine cells that migrated toward cancer cells even before invasion in pancreatic cancer. After contacting with SCs, pancreatic cancer cells move along neurites, during which physical contact and cellular signals play significant roles (21). Previous studies have reported that SCs secreted soluble factors including chemokine and adhesion molecules, affecting pancreatic cancer cells movement (22–24); however, no studies to date have explored the potential role of SCs in cervical cancer.

In this study, we explored the role of CCL2, identified its association with SCs and elucidated its potential clinical value in predicating PNI and selecting the appropriate surgical method for cervical cancer.

## Materials and Methods

### Gene Set Enrichment Analysis

All raw genomic data and clinical data were obtained from the GEO (Gene Expression Omnibus) database. We obtained four RNA sequence datasets with the keyword “perineural invasion” and “tumor” (GSE103479, GSE86544, GSE102238 and GSE7055). Gene Set Enrichment Analysis (GSEA) was performed to seek common pathways of different cancers using PNI as the criteria for sample classification.

#### Cell Cultures

All cell lines were purchased from the Cell Bank of the Chinese Academy of Sciences (Shanghai, China), including HeLa, ME-180, SiHa, CaSki, C33A, MS751, and HCC94. The human cervical adenocarcinoma (AC) cell line HeLa, Rat Schwann cell line (RSC96), SiHa, C33A, MS751 were cultured in Dulbecco's modified Eagle's medium (DMEM) (Gibco, Carlsbad, CA, USA), supplemented with 10% fetal calf serum (FCS) (Invitrogen, CA, USA). The human cervical squamous cell carcinoma (SCC) cell line ME-180 was grown in Mycoy'5A medium containing 10% FCS. CaSki and HCC94 were grown in RPMI-1640. DRGs were dissociated from the female Sprague Dawley rats. All cells were incubated in a 5% CO_2_-humidified incubator at 37°C.

### Cell Migration and Invasion Assays

Cell migration and invasion assays were performed using 8.0 μm pore transparent polyethylene terephthalate inserts (Corning Inc., Glendale, AZ, USA) in 24-well plates. 1 × 10^5^ cells in 0.2 mL of FCS free media were added to each of the inserts, with or without Matrigel, while DRG or RSC were placed in the bottom with 0.6 ml of medium supplemented with 10% FCS. After 20 h for migration assay and 36 h for invasion assay, the membranes were fixed with 4% polyoxymethylene at room temperature for 30 min and then stained with crystal violet staining solution (YEASEN, Shanghai, China) for 30 min. Five random fields were counted at ×10 magnification. The cells for each membrane were quantified by counting five random fields at ×20 magnification.

### Wound Healing Assay

Cancer cells were cultured with complete medium until 80% confluence in 6-well plates. Then slowly scratch the monolayer across the center of the well with a 200 μl pipette tip. After washing twice, fresh medium was added into the well. Images of a scratch were captured at a 0, 24, and 48 h and processed using Image J software.

### *In vitro* Neural Invasion Assay

A Matrigel/DRG model *in vitro* was constructed by Huyett et al. (25) and was frequently used to investigate the interaction between nerve cells and cancer cells. DRG are harvested from the spinal column of a sacrificed Sprague Dawley rat and placed in the center of 2.5 μl of matrix. Cancer cell lines were placed peripherally around the matrix 2 days later. Cellular movement was detected by confocal microscopy at a 24 h interval.

### Western Blotting

Protein lysates were resolved by electrophoresis on SDS-PAGE, and proteins were transferred to NC membrane. After blocking in 5% non-fat milk in 1 × TBST for 1 h, the membranes were incubated at 4°C overnight with primary antibodies including CCR2 (12199S, Cell Signaling Technology), MCP1 (ab25124, Abcam), MMP9 (ab76003, Abcam), MMP2 (ab92536, Abcam), MMP12 (ab52897, Abcam) and β-actin (CST-5174T, Cell Signaling Technology), and EMT Antibody Sampler Kit (CST-9782, Cell Signaling Technology). The antibodies were diluted as recommended by the manufacturers.

### Histological Analysis

The acquisition protocol was approved by the Institutional Ethics Committee of the International Peace Maternity and Child Health Hospital (IPMCH). Twenty samples with PNI and 36 samples without PNI collected between 2012 and 2018 were utilized in this research. These tissues were embedded in paraffin and then cut into 4 μm sections. The sections were stained with haematoxylin & eosin (H&E). For immunohistochemical assay, sections were incubated with a CCR2 antibody at 4°C overnight followed by secondary antibody conjugated with HRP. The images were obtained by microscopy (Leica, Germany). The positive nerve fibers were counted in a blinded fashion in 10 representative fields.

The tissue sections from mice were incubated with primary antibodies including CCR2 (bs-0562R, Bioss), N-cadherin (ab18203, Abcam), E-cadherin (3195T, Cell Signaling Technology), Snail (bs-1371R, Bioss), and Slug (bs-1382R, Bioss) followed by the same procedures described above.

### Real-Time PCR

Total RNA was isolated using TRIzol Reagent (Invitrogen, CA, USA), and cDNA was synthesized using the cDNA Synthesis SuperMix kit (TransGen Biotech Co., Beijing, China). The real-time PCR was performed using quantstudio 7 flex system. The resulting data were normalized to housekeeping genes GAPDH. The primers used for the amplification were as follows: for CCL2-Forward (5′-accactatgcaggtctctgtca-3′) and CCL2-Reverse (5′-ggcattaactgcatctggctga-3′), GAPDH-Forward (5′-catggcctccaaggagtaag-3′) and GAPDH-Reverse (5′-ggtctgggatggaattgtga-3′).

### Flow Cytometry

The HeLa or ME-180 cells were incubated in 1 mL of diluted CCR2 (357208, Biolegend) and Ki67 antibody (CST-9449S, Cell Signaling Technology) on ice for 30 min after being harvested, fixed, washed, and blocked. Then, secondary antibodies conjugated with Alexa Fluor®488 and Alexa Fluor®594 were added into the buffer and the samples were measured by FACS Calibur flow cytometry (BD, NJ, USA). Data were processed by FlowJo software (LLC, Ashland, USA).

### Immunofluorescence Assay

For identification of cancer cells and DRGs, the cells were fixed with 4% paraformaldehyde for 1 h and blocked with 0.03% Triton X-100 containing 5% calf serum in PBS for 1 h at room temperature and then incubated with the antibody pan-cytokeratin (CST-4544, Cell Signaling Technology), PGP9.5 (PA5-16825, ThermoFisher), Neurofilament-heavy (PA3-16753, ThermoFisher), and Collagen IV (ab6586, Abcam) at 4°C overnight. Then, the cells were incubated with a secondary antibody conjugated with Alexa Fluor®488 (A-11006, ThermoFisher) and Alexa Fluor® 594 (A-11007, ThermoFisher).

For immunocytochemistry, the cells were fixed, washed, blocked, and incubated with primary antibodies against pan-cytokeratin or CCL2 (ab9899, Abcam) overnight at 4°C. Staining was detected with Alexa Fluor®594. The cells were counterstained with DAPI.

The slides from mice were incubated with MMP9 (10375-2-AP, Proteintech) and MMP2 (10373-2-AP, Proteintech) and then a secondary antibody conjugated with Alexa Fluor®594 was added in. The images were obtained by fluorescence microscope (Leica, Germany).

### ELISA Assay

Serum samples from 15 normal cervical samples and 33 cervical cell carcinoma samples were obtained from IPMCH. Besides, cell culture media under different conditions were collected and prepared. The detailed procedures were conducted in accordance with the protocol in the MCP-1 immunoassay kit (EK0902, BOSTER).

### Lentivirus Infection

HeLa and ME-180 cells were implanted in the 6-well plates at appropriate densities. The short hairpin RNAs (shRNA) against CCR2 (sh7732, sh7733, sh7734) and the negative control shRNA were obtained from Obio Technology (Shanghai, China) and were added into the wells. Puromycin was used to select stable transfected populations of cells.

### *In vivo* Perineural Invasion Assay

The sciatic nerves were surgically exposed after anesthetizing athymic nude mice with isoflurane. The mice were grouped randomly into five groups (PBS, HeLa, ME-180, HeLa shNC, and HeLa shCCR2) of 9 mice each. 3 × 10^5^ cells of every type in 3 μL PBS was injected into the distal sciatic nerve under the epineurium using a 10 μL Hamilton syringe. Five weeks later, the mice were euthanized to observe the metastasis and excise their sciatic nerves to measure PNI-related indexes. Then these sciatic nerves were cut into 8-μm-thick sections. This study was approved by the Institutional Ethics Committee of the International Peace Maternity and Child Health Hospital (IPMCH), number: [GKLW] 2017-125.

### FITC-Phalloidin Staining and CellTracker CM-DiI Labeling

FITC-phalloidin staining was performed to detect the microfilaments and cytoskeletal reorganization insides the cells. After being washed, cells were fixed in 4% formaldehyde at room temperature for 15 min and permeabilized using 0.1% Triton X-100 in PBS for another 10 min. Cancer cells were incubated with FITC-phalloidin working solution (C1033, Beyotime) for 1 h. Cells were counterstained with DAPI.

ME-180 cells were incubated with red fluorescent CM-DiI (40718ES50, YEASEN) for 30 min, and then the CM-DiI was washed with PBS. All images were captured via confocal microscopy.

### Statistical Analysis

Data was processed using GraphPad Prism 7 software and presented as mean ± standard error of mean (SEM). Comparisons between different groups were using two-tail unpaired Student's *t-*test or one-way analysis of variance (ANOVA). Differences were considered significant if the *P* < 0.05.

## Results

### The Occurrence of PNI in Cervical Cancer

**Figure 1:**
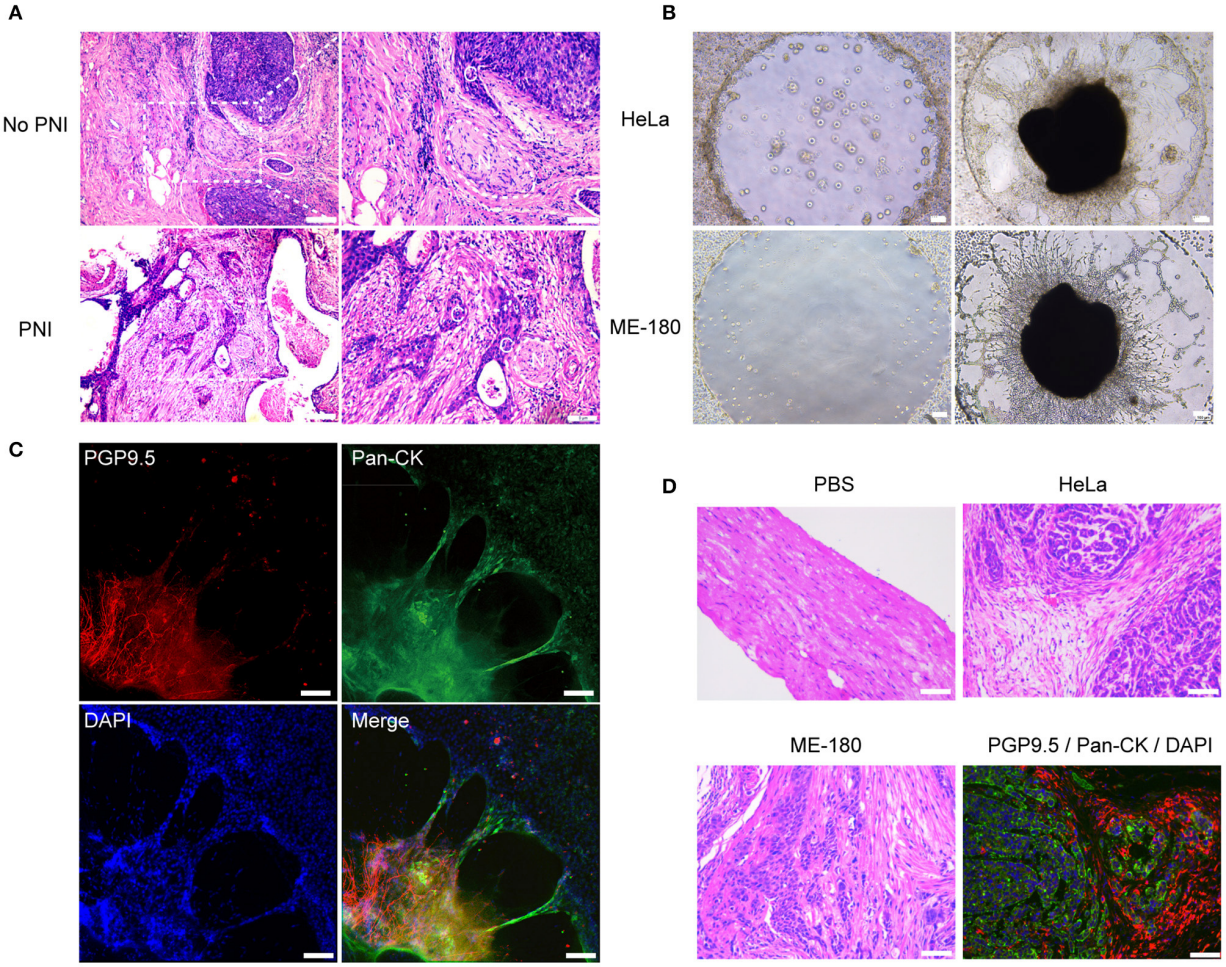
The presence of perineural invasion *in vitro* and *in vivo*. **(A)** Hematoxylin and eosin (HE) stained image of non-PNI and PNI cervical cancer tissues. The area of nerves is marked by a dashed line. N refers to nerves and C refers to cancer cells. **(B)** Cervical cancer cell lines, HeLa and ME-180, induced PNI in an *in vitro* model. DRG was placed in the center of Matrigel (50× magnification, scale bar, 100 μm). **(C)** Double immunofluorescence staining of neurites and HeLa cells in the perineural niche. Staining: PGP9.5, pan-cytokeratin (pan-CK), DAPI, and overlay respectively (100× magnification, scale bar, 100 μm). **(D)** H&E stained image of sciatic nerves injected with PBS, HeLa and ME-180, respectively. Image in the right refers to double immunofluorescence staining of sciatic nerve (PGP9.5) and HeLa cells (pan-CK) (200× magnification, scale bar, 50 μm).

Perineural invasion, a phenotype that cancer cells invade into the perineural space of local peripheral nerves and contact to the endoneurium directly, could be observed in specimens of cervical cancer (Figure 1A). We assessed the potential of PNI in seven cervical cancer cell lines including HeLa, ME-180, SiHa, CaSki, C33A, MS751, and HCC94 by cocultivation with DRG separately. After 2 days of cocultivation, HeLa and ME-180 cells showed their notable ability to interact with DRG neurites, whereas the other five cell lines had little or no interaction with DRG (Supplementary Figure 1A). We counted the contact of neurites and cancer cell clusters after 3 days of cocultivation (Figure 1B, Supplementary Figure 1B). HeLa and ME-180 were prone to PNI, SiHa, and CaSki had inferior abilities to interact with nerve cells. In contrast, C33A, MS751, and HCC94 demonstrated low contact with neurites. Confocal imaging of neurites stained with the pan neuronal marker PGP9.5 and cancer cells stained with pan-cytokeratin (pan-CK) revealed the close touch between the DRG and HeLa cells. After 3 days of cocultivation, HeLa cells had already arrived at the DRG in the center of Matrigel along the neurites (Figure 1C). Marking ME-180 cells with CellTracker CM-DiI and staining neurites with neurofilament-heavy (NF-H) antibody, we found that ME-180 cells could also spread along neurites toward DRG (Supplementary Figure 1C).

In order to further analyze the frequency of PNI of cervical cancer cells, HeLa and ME-180 cells were injected into the mouse sciatic nerves. Four of nine and three of nine mice injected with HeLa and ME-180 cells, respectively showed perineural invasion. Cancer cells colonized and spread along the nerve fibers, which were validated by H&E staining of sciatic nerves and double immunofluorescent staining of PGP9.5 and pan-CK (Figure 1D). These results showed the occurrence of PNI from different levels.

### The Interaction Between Nerve Cells and Cervical Cancer Cells

**Figure 2:**
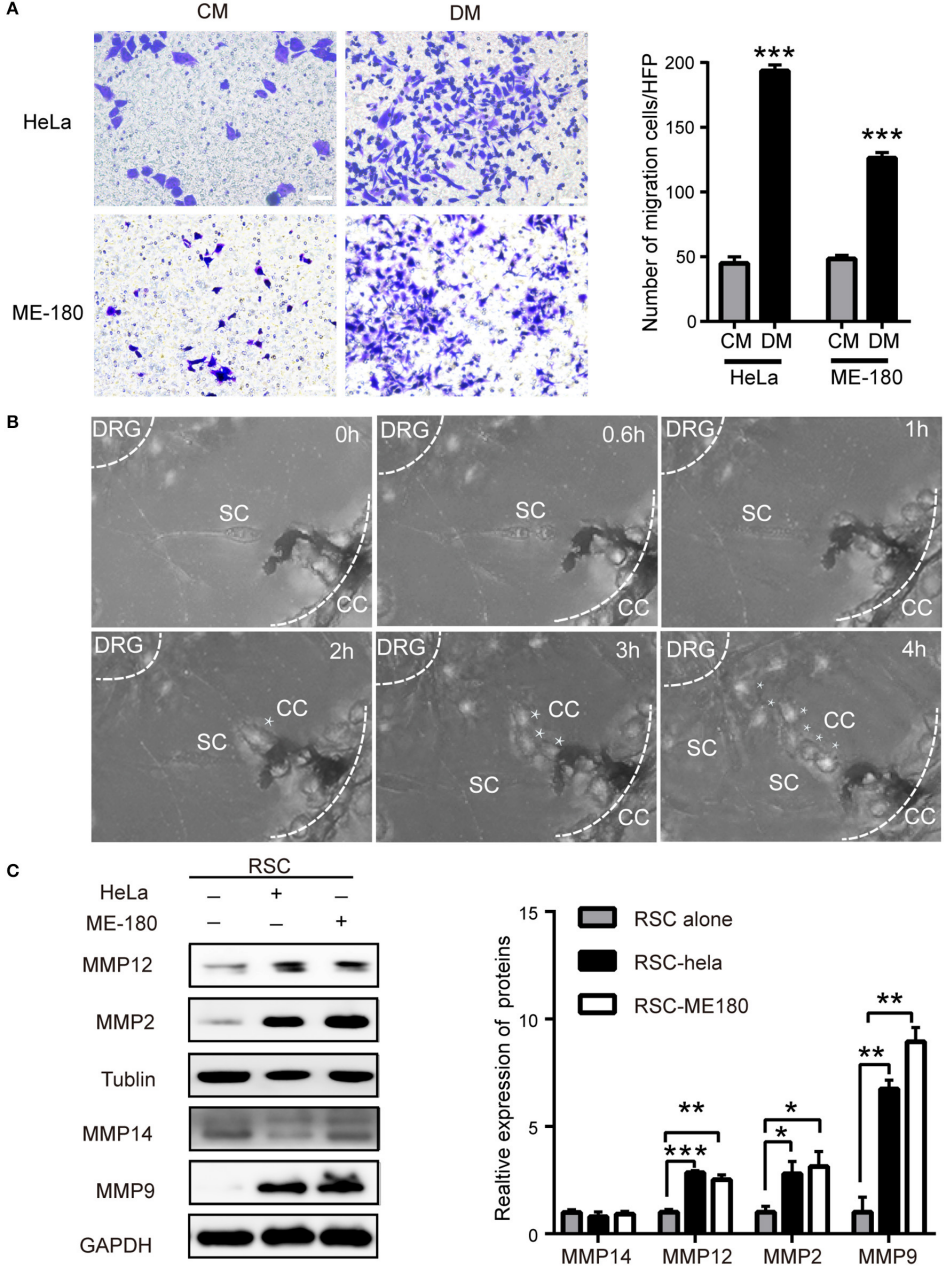
The crosstalk between SCs and cervical cancer cells. **(A)** DM promotes cell migration of HeLa and ME-180 cells compared with CM. DM, DRG medium; CM, Control medium. (**B)** Cocultivation of DRGs with HeLa cells. Images from confocal microscopy shows the process of a cancer cell (marked by asterisks) toward the center of the DRG. Assume that the time of the first picture is 0 h. The SCs are marked by white arrows. The smaller white dotted lines describe the border of the DRG and the larger defines the Matrigel edge. **(C)** HeLa and ME-180 cells upregulated the expression of MMP2/9/12 in SCs. *P < 0.05, **P < 0.01, *** *P* <0.001.

The signals in the DRG medium (DM) markedly enhanced the migration and invasion of HeLa and ME-180 cells (Figure 2A, Supplementary Figure 2A). In turn, the continuous confocal recording over a 12 h period indicated that SCs were firstly triggered and moved toward HeLa cells before the onset of cancer invasion. Upon contact, SCs induced HeLa cell dispersion and movement directionally toward the DRG (Figure 2B). Coculturing with ME-180 cells, SCs were activated and arrived at the sites of cancer cells (Supplementary Figure 2B). There was some difference that SCs linked with each other and more time was needed to induce ME-180 cells movement comparing to HeLa cells. Based on these observations, SCs as primary, non-neoplastic cells were activated, arrived at the sites of cancer cells, attracting cancer cells spread toward DRGs.

Rat Schwann cells (RSC96) were identified with s100β antibody and used in experiments (Supplementary Figure 3A). PNI is involved in SCs and cancer cells motility, which requires an adaptable microenvironment to degrade the ECM. ECM integrity is regulated by the balance between matrix metalloproteinases (MMPs) and their inhibitors (26). In the co-culture supernatant, the concentration of MMP9 markedly increased (Supplementary Figure 3B). SCs displayed increased expression of MMP-2, MMP-9, and MMP-12 at the protein level after co-cultivation with cervical cancer cells, whereas no change in the expression of these MMPs was detected in HeLa and ME-180 cells, differing from previous report about pancreatic adenocarcinoma cells (23) (Figure 2C, Supplementary Figure 3C). Immunofluorescent analysis also displayed the increased expression of MMP9 and MMP2 of sciatic nerves after being injected with HeLa cells or ME-180 cells (Supplementary Figure 3D). The metalloproteases, especially MMP2 and MMP9 were reported to degrade type IV collagen (27). Therefore, we detect the expression of type IV collagen in both the PNI and non-PNI sites (Supplementary Figure 3E). Cancer cells next to a peripheral nerve had a lighter staining of type IV collagen than that distant from nerves. These results indicated that SCs and cervical cancer cells interacted with each other.

### The CCL2/CCR2 Axis Enhances the Proliferation, Migration, and Invasion of Cervical Cancer Cells

**Figure 3:**
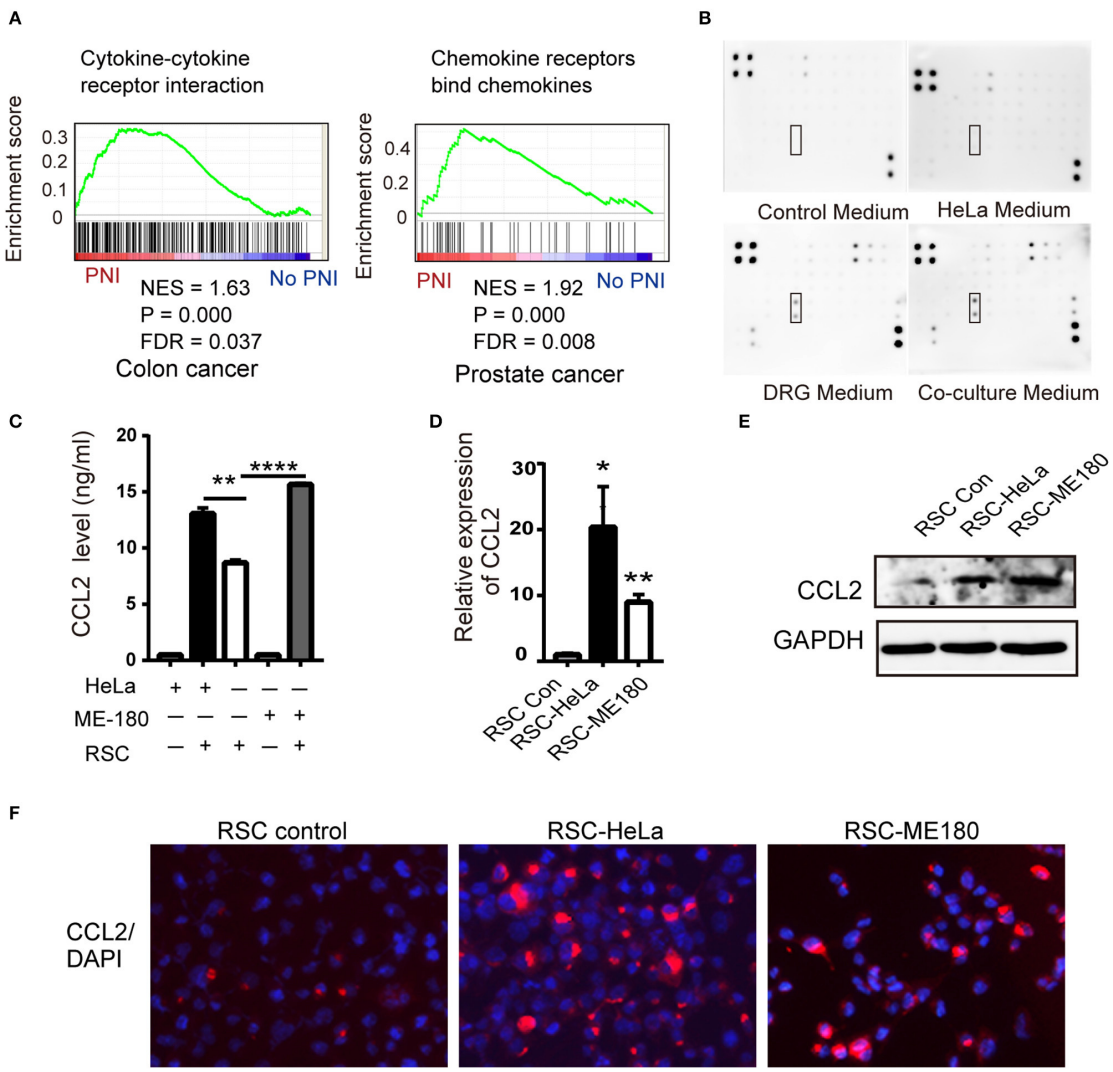
CCL2 is expressed in DRGs and SCs. **(A)** GSEA results shows the correlation of PNI and cytokine and cytokine receptor interaction in colon cancer as well as PNI and the chemokine receptor in prostate cancer. NES normalized enrichment score, FDR false discovery rate. **(B)** Cytokine array of DRG conditioned medium, HeLa conditioned medium, coculture medium of DRG and HeLa, with DMEM media as a control, identifying the expression of CCL2. **(C–F)** ELISA, RT-PCR, WB, and immunofluorescence analyses revealed that the CCL2 expression of RSC increased after cocultivation with HeLa and ME-180 cells (200× magnification, scale bar, 50 μm). *P < 0.05, **P < 0.01, ****P < 0.0001.

To identify common pathways involved in PNI, the raw gene expression data of several PNI-related cancers including colon cancer, head and neck cancer, pancreatic ductal adenocarcinoma, and prostate cancer (GSE103479, GSE86544, GSE102238, and GSE7055) were downloaded and conducted GSEA. Cytokine receptor interaction was selected for further study not only for its significant *P*-value in colon cancer and prostate cancer (Figure 3A), but also the enrichment in cytokine production and cytokine-mediated signaling pathway in head and neck cancer patients diagnosed with PNI (28). A cytokine array kit was used to screen molecules involved with PNI and CCL2 was identified as the target among 34 cytokines for its upregulation in cocultivation group (Figure 3B). The monocytes recruited by CCL2 were found to mediate PNI of pancreatic cancer (17). CCL2 was mainly produced by DRGs and the levels of CCL2 increased after coculturing ME-180 cells and DRGs (Supplementary Figure 4A). SCs upregulated CCL2 after nerve injury, which then attracted macrophages into the nerves (29). To identify the source of CCL2 during PNI, we assessed the expression of CCL2 in SCs. After cocultivation with HeLa and ME-180 cells, the expression of CCL2 in SCs and its receptor CCR2 on the membrane of cancer cells both increased (Figures 3C–F, Supplementary Figure 4B).

**Figure 4:**
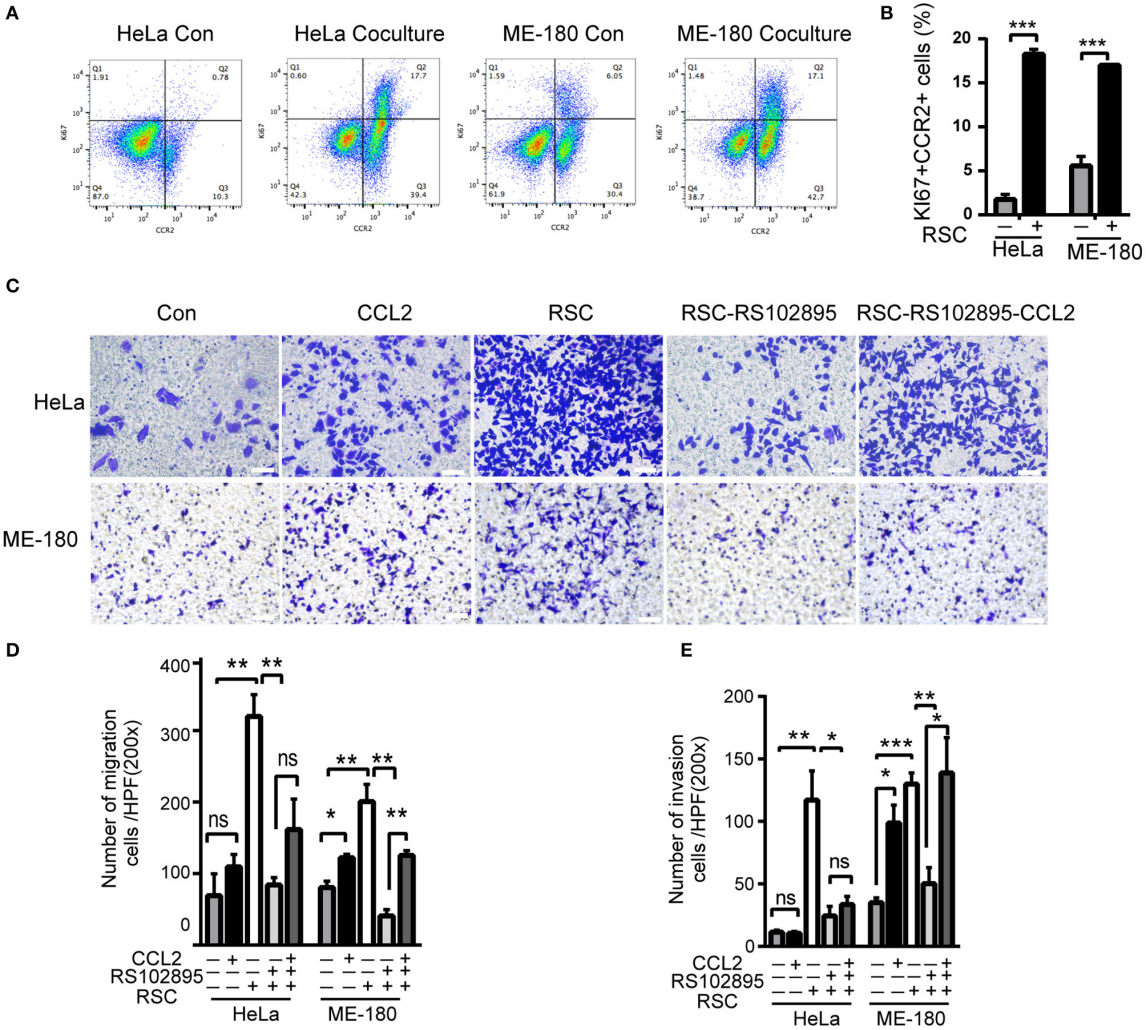
CCL2 promotes cervical cancer proliferation, migration, and invasion. **(A,B)** Flow cytometry results of phagocytosis assays in which HeLa or ME-180 cells were cocultured with or without SCs. The percentages of Ki^+^ CCR2^+^ cells are indicated in the upper right corner. (**C–E)** Cell migration and invasion assays were performed to assess the effect of RSC, CCL2, and CCR2 on cancer cells. The data are shown as the mean ± SEM. *P < 0.05, **P < 0.01, ***P < 0.001 and ns, not significant. **(D,E)** Refer to the statistical results.

We next used flowcytometry to detect HeLa and ME-180 cells marked by CCR2 and Ki67 antibodies, and found significant increases in the proportion of CCR2^−^ KI67^+^, CCR2^+^ KI67^−^, and CCR2^+^ KI67^+^ cells in the co-culture group (Figures 4A,B). The majority of CCR2^+^ positive cells were in the proliferative phase, which means that SCs might affect cellular proliferation through CCR2. CCL2 at different concentrations (50, 100 ng/ml) resulted in a rise in the wound healing percentages of ME-180 cells, suggesting CCL2 as a chemoattractant of ME-180 cells (Supplementary Figures 4C,D). To investigate whether CCL2 is required for movement of HeLa and ME-180 cells, we added CCL2 and CCR2 antagonist, RS102895 into the lower layer of the Boyden chamber as required. The ectopic CCL2 stimulation (50 ng/ml) promoted the migration and restored the mobility interrupted by RS102895 of ME-180 cells rather than HeLa cells, whereas RS102895 could reduce the number of SCs-induced HeLa and ME-180 cells (Figures 4C–E). These results reveal that CCL2 plays an important role in inducing cervical cancer cells movement toward nerve cells.

### CCL2 Induces Cervical Cancer Cells EMT by Binding CCR2

**Figure 5:**
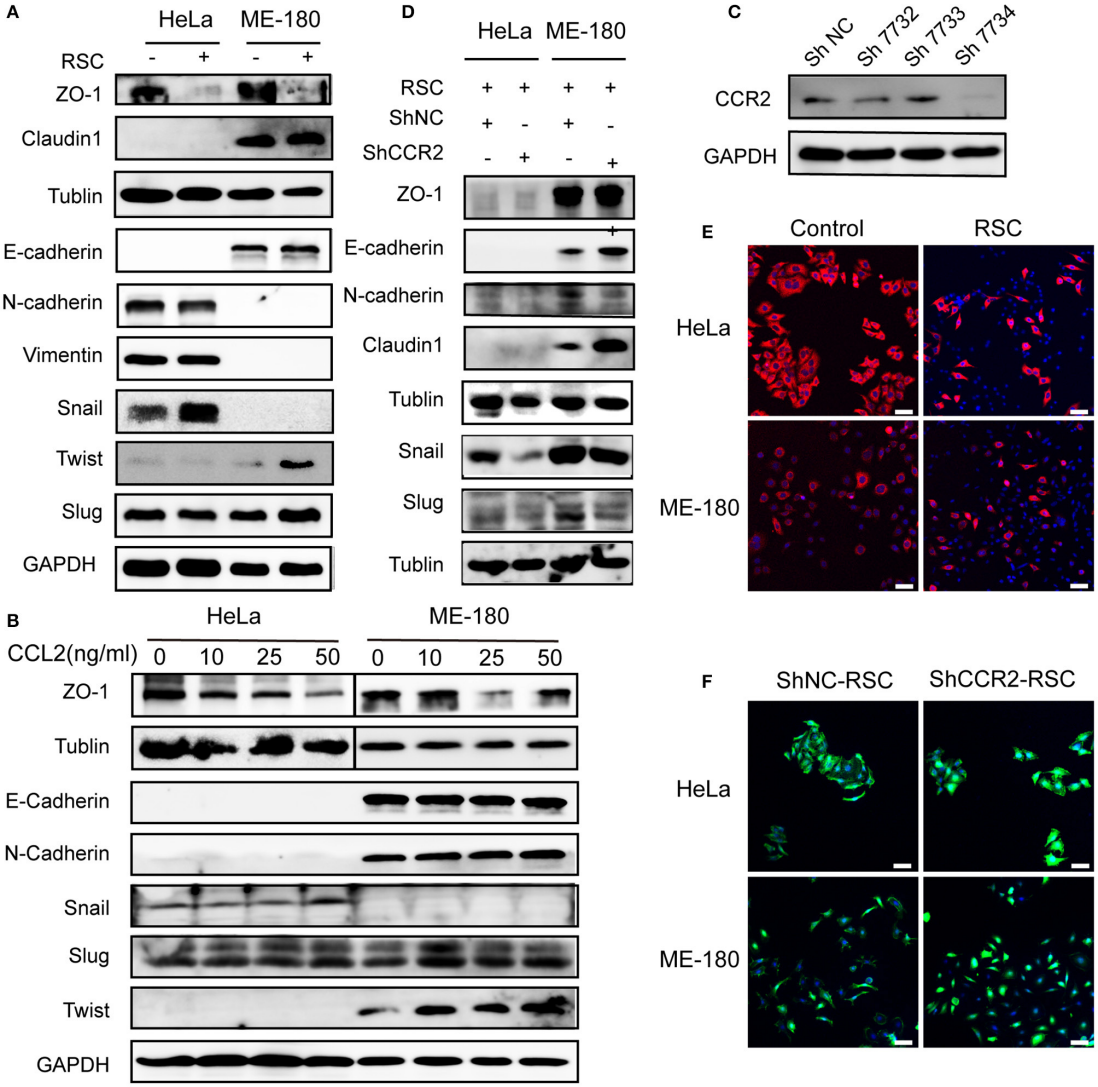
Effect of CCL2 on EMT of human cervical cancer cells. **(A)** Markers of EMT were altered in HeLa and ME-180 cells after cocultivation with SCs. GAPDH or β-tublin were used as internal loading controls. **(B)** Hela and ME-180 cells were exposed to different concentrations of CCL2 followed by protein isolation, to assess EMT markers via western blot. **(C)** Expression of CCR2 was measured in HeLa cells after transfection with a panel of shRNAs targeting CCR2 or control shRNA. The sh7734 was used to create stably silenced shCCR2 cells. **(D)** Western blot analyses of the EMT related markers after silencing CCR2 in HeLa and ME-180 cells. **(E)** SCs brought about morphological changes ranging from an epithelial plasticity toward a fibroblast-like phenotype (stained with pan-CK) (100× magnification, scale bar, 100 μm). **(F)** Downregulation of CCR2 in HeLa and ME-180 cells showed opposing changes through FITC-phalloidin staining compared with the control group (100× magnification, scale bar, 100 μm).

EMT is regarded as a key step during the cancer progression from primary site toward metastasis (30). SCs induced the downregulation of ZO1 and the upregulation of Snail in HeLa cells, while SCs mainly upregulated the expression of Slug and Twist as well as downregulated the expression of ZO1 in ME-180 cells (Figure 5A). As shown in Figure 5B, CCL2 at the concentration of 50 ng/ml stimulating HeLa cells increased the expression of Snail and decreased the expression of ZO1 at the protein level. In contrast, both 25 and 50 ng/ml of CCL2 decreased the expression of ZO1 and 10 ng/ml of CCL2 increased the expression of Slug and Twist in ME-180 cells. Transfection of CCR2 shRNA significantly reduced CCR2 expression (Figure 5C). The EMT process were nearly reversed after interfering with the expression of CCR2, indicating that the CCL2-induced EMT was CCR2 dependent. A slight difference in the expression of E-cadherin, N-cadherin and Claudin-1 was also found in ME-180 cells (Figure 5D).

HeLa and ME-180 cells both showed morphological changes after co-cultivation with SCs, becoming slenderer with a fibroblast-like appearance (Figure 5E). FITC-phalloidin staining was performed to detect the cytoskeleton. ShRNA-mediated CCR2 interference caused the collapse of the cytoskeletal organization and reduced the pseudo foot formation compared to the shNC group in both cell lines (Figure 5F).

### CCL2/CCR2 Contributed to PNI and Induced EMT of Cervical Cancer *in vivo*

**Figure 6:**
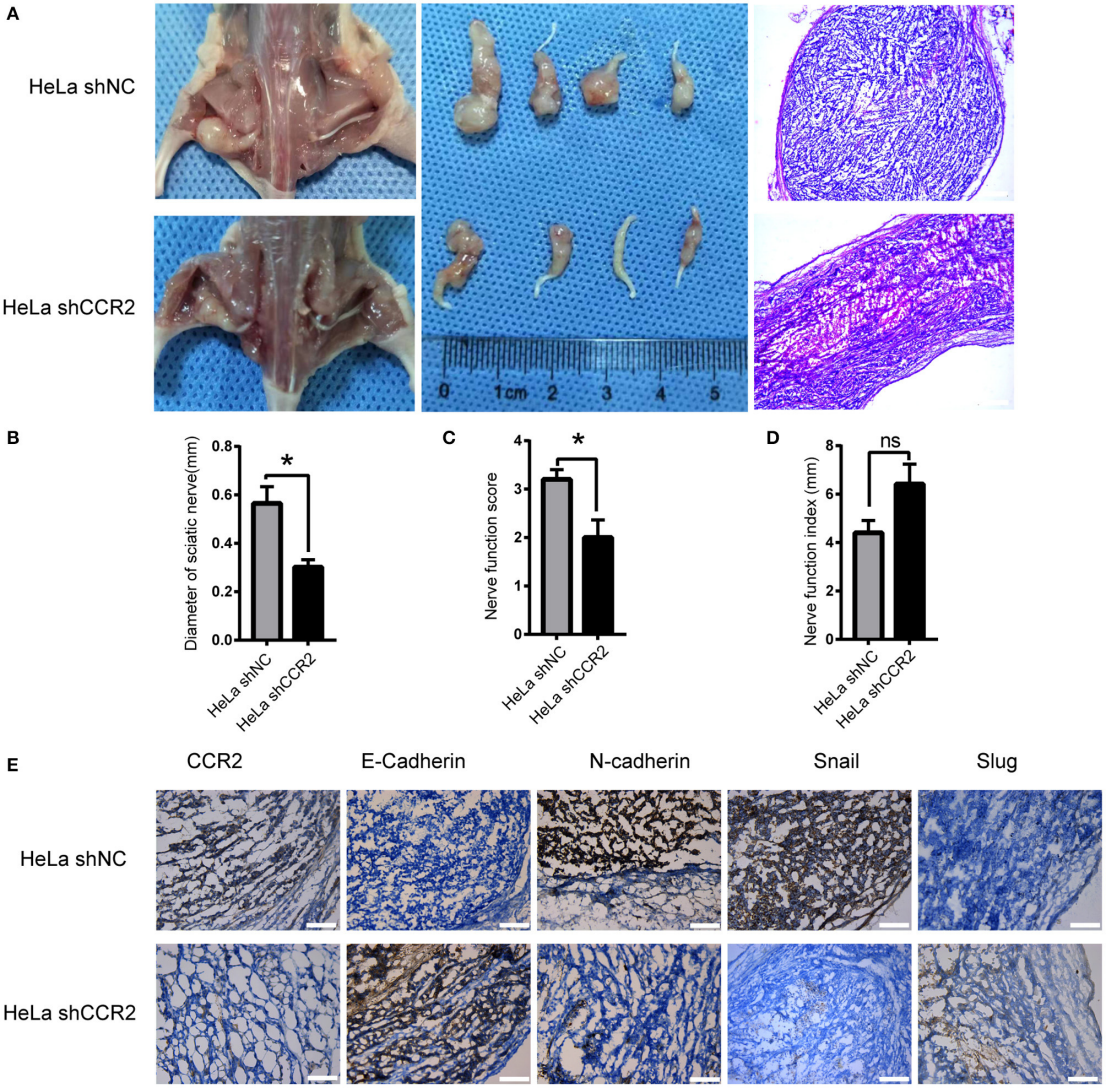
Relationship between CCL2/CCR2 axis and PNI of cervical cancer *in vivo*. **(A)** Surgical images of the sciatic nerve. HeLa cells were injected into the left sciatic nerves. Mice treated with HeLa shCCR2 cells exhibited a smaller volume. The images to the right refer to the corresponding H&E staining results (50× magnification, scale bar, 200 μm). **(B–D)** Diameter of sciatic nerves, sciatic nerve function score and sciatic nerve function index of mice. **(E)** HeLa shNC cells injection resulted in the upregulation of N-cadherin and Snail. HeLa shCCR2 cells injection resulted in highly positive E-cadherin staining. *P < 0.05, *ns*, no significant. (200× magnification, scale bar, 100 μm).

The ability of CCR2 blocking PNI *in vivo* was assessed using a neural invasion model with balb/c nude mice. HeLa shNC and HeLa shCCR2 cell suspensions were injected into the sciatic nerves in the left. Five weeks after injection, 4 of 9 mice in two groups showed perineural invasion and the sciatic nerve scores and sciatic nerve index of the left hind limb were assessed. In the shCCR2 group, the sciatic nerve score was dramatically decreased compared with the shNC group and accompanied by an increase in the sciatic nerve function index (Figures 6C,D). Following euthanasia, the tumors were resected for histologic examination (Figure 6A). The tumor diameters at 2, 4, and 6 mm from the implantation site were measured the mean values of which were regarded as the sciatic nerve diameter. The diameter of sciatic nerve in shNC group was significantly larger compared to the shCCR2 group (Figure 6B), suggesting that the CCL2-CCR2 axis mediated the progression of cervical cancer cells along the sciatic nerve.

Next, we analyzed the expression of CCR2 and EMT-markers including E-cadherin, N-cadherin, Slug, and Snail. After CCR2 was silenced, the expressions of Snail and N-cadherin decreased and E-cadherin increased (Figure 6E). These results demonstrate that blocking the CCL2/CCR2 signal pathway could inhibit cervical cancer growth and decrease the invasion distance along the sciatic nerve.

### Expression and Clinical Significance of CCL2/CCR2 in Cervical Cancer

**Figure 7:**
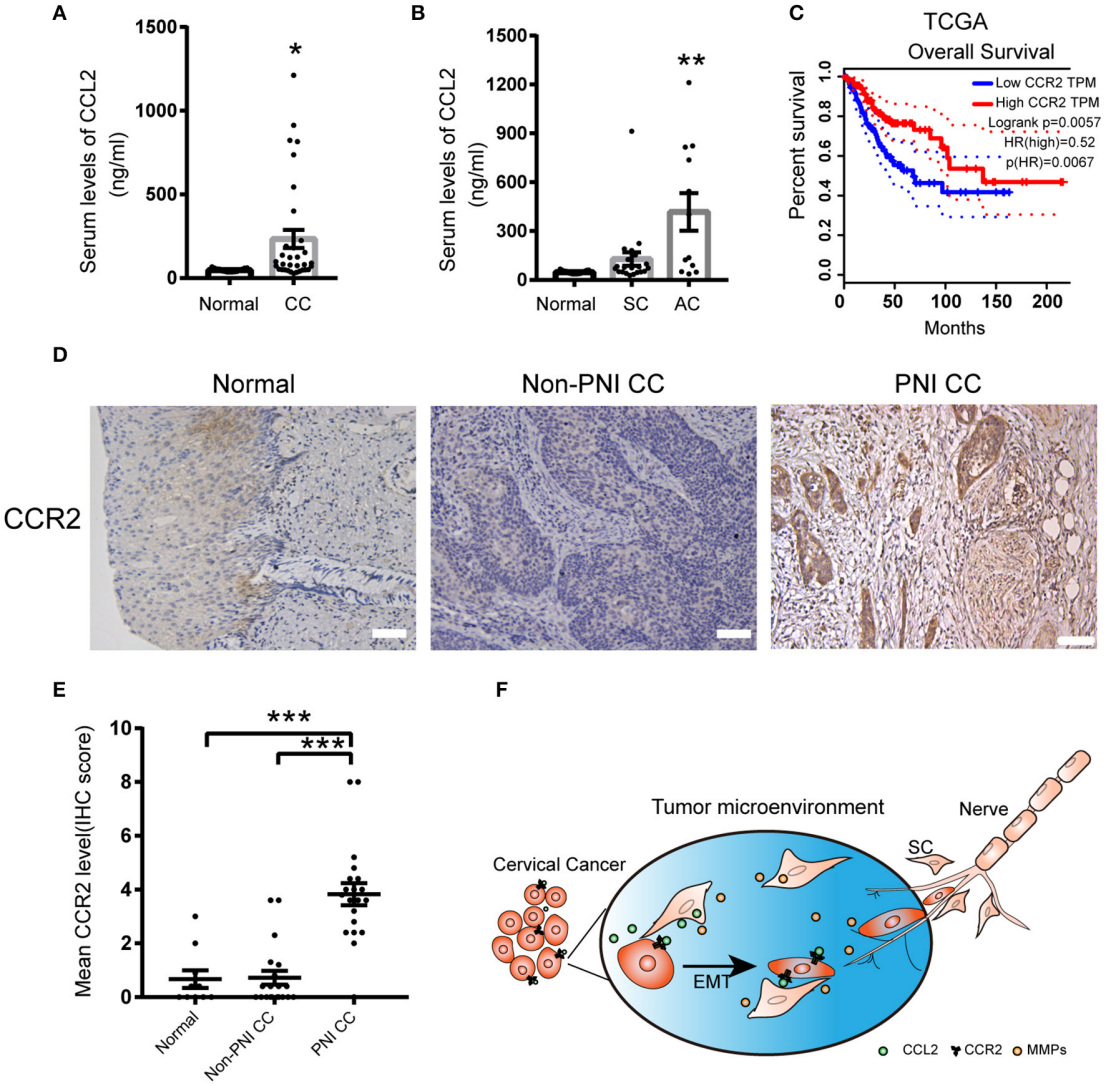
The expression of CCL2 and CCR2 in cervical cancer samples. **(A)** CCL2 levels in the serum of 15 normal samples and 33 cervical cancer samples detected by ELISA. **(B)** CCL2 levels in the serum of cervical adenocarcinoma samples upregulated compared to normal samples (*P* = 0.0014). **(C)** Public data shows that CCR2 mRNA expression levels are negatively correlated with the prognosis of cervical cancer (*P* = 0.0057). **(D,E)** CCR2 staining in 20 cervical cancer samples with PNI was stronger than cancer samples without PNI (*n* = 20) and normal cervical samples (*n* = 16) (200× magnification, scale bar, 50 μm). *P < 0.05, **P < 0.01, ***P < 0.001 compared to the normal group. ns, not significant. **(F)** Schematic showing the mutual interaction between SCs and cervical cancer cells.

The serum values of CCL2 in normal cervical samples and cervical cell carcinoma were 48.33 ± 2.618 (pg/ml) and 233.7 ± 54.26 (pg/ml), respectively (Figure 7A). Due to a fluctuating range in CCL2 values, we further subdivided the tumor type to cervical SCC and AC. The levels of CCL2 in the serum of cervical AC samples were significantly upregulated compared to normal samples (417.9 ± 115.5, *P* = 0.0014), whereas no significant change in the CCL2 values was detected between normal samples and cervical SCC, which might indicate that CCL2 is a suitable marker of PNI in cervical AC (Figure 7B).

The results obtained from the cancer genome atlas (TCGA) showed that enhanced expression of CCR2 might correlate with poor overall survival in human cervical cancer (Figure 7C). IHC staining of CCR2 protein was performed on tissue sections from 16 human normal cervical samples, 20 cervical non-PNI cancer samples, and 20 with PNI. The expression of CCR2 was increased in the samples with PNI compared to the non-PNI and normal groups (Figures 7D,E). These data suggested that the detection of CCL2/CCR2 might contribute to early detection of PNI in cervical cancer.

## Discussion

PNI is gradually paid more attention to with its clinical significance. Neoplastic cells infiltrating into the perineurium space were spared by tumor resection, leading to local recurrence (31). PNI has been reported as an important danger factor for independent survival, indicating poor prognosis for many malignancies, including prostate cancer (32–34), colon cancer (35–37), head and neck cancer (38), gastric cancer (39), and cervical cancer (40). In cervical cancer, PNI could be considered as an indication guiding the operation and postoperative adjuvant therapy (41). Nerve-sparing radical hysterectomy (NSRH) preserves the pelvic autonomic nerves, thus leading to a much improved quality of life, comparing with Radical hysterectomy (RH) (42, 43). Given the association between PNI and poor prognosis of cervical cancer patients, an appropriate marker of PNI is in an urgent need. Our results demonstrated that the CCL2/CCR2 axis played a crucial role in PNI and promised to be a marker guiding the selection of NSRH.

Patients with AC had a worse survival compared to SCC (44, 45). Our results found that the serum CCL2 levels were increased in cervical cancer samples, especially in patients with AC. Moreover, the CCL2/CCR2 axis mediated PNI of cervical cancer *in vitro* and *in vivo*. Previous studies have reported that the CCL2 mRNA expression in cervical carcinoma cells were related to local recurrence and distant metastasis significantly and the absence of CCL2 expression indicated an increased survival (46). The upregulation of CCL2 in the serum of patients of AC may partially explain its poor prognosis in contrast with SCC and detailed mechanism needs further elaboration.

Chemokines contribute to the mechanisms underlying PNI. The chemokine CCL2, not only recruits immune cells including monocytes, dendritic cells, natural killer (NK) cells memory T cells and dendritic cells (47), but also promotes changes in neuronal excitability and nerve repair (48). CCL2 and its receptor CCR2 were reported to play crucial roles in cancer metastasis, accelerate cancer cell growth, and promote their colonization at metastasis sites (49). CCL2 is mainly secreted by SCs to attract macrophages in response to nerve injury (50). The expression of CCR2 could be detected in the membrane of cancer cells (51, 52). Our results indicated that the migration and invasion of HeLa and ME-180 cells were both significantly increased after co-cultivation with SCs, which could be interrupted by a CCR2 antagonist. CCL2 also influenced cellular proliferation and morphological changes to support cervical cancer cell migration toward SCs. Blocking CCR2 induced the collapse of the cytoskeletal organization and mesenchymal to epithelial transition *in vitro* and *in vivo*. These results identified CCL2 as a chemotactic factor attracting cancer cells toward nerves and ultimately causing their metastasis along the neurites.

PNI is the result of an active crosstalk between nerves and cancer involving in many molecules, resulting in neuron outgrowth and fueling tumor progression (12, 53, 54). We found that SCs could arrive at cervical cancer cell sites and then induce them metastasis. Meanwhile, we detected high expression of CCL2 in SCs, while cervical cancer cells expressed lowly. Our study demonstrated that SC-derived CCL2 modulated the PNI of cervical cancer. As the observation of the movement of SCs, there must be signals derived from cervical cancer cells attracting SCs movement. In a recent study, CXCL12 secreted by cancer cells bound to its receptors and attracted receptor-positive SCs to pancreatic cancer cells, thereby initiating PNI (55). A similar effect on nerves by cancer cells were reported in the brain metastasis of breast cancer. Microglia emerged at the site of breast cancer and enhanced the invasion of cancer cells into the brain and then spread away when co-cultured with breast cancer cells (56). The signals from cancer cells during PNI require further investigation.

As endopeptidases, the MMP family plays a role in ECM degradation and tissue remodeling (11). MMP2 and MMP9 belong to the gelatinase subgroup of MMPs, which degrade type IV collagen (57). MMP12 is a proteinase mainly secreted by macrophages and inhibits inflammation through regulation of the CCL2/CCR2 signal axis (58). A previous study reported that MMP2 and MMP9 were mainly secreted by pancreatic cancer cells to modulate the neural cancerous microenvironment (23). SCs could also secret MMP2 and MMP9 to support axonal regeneration (59). Here, we confirmed that cervical cancer cells upregulated the expression of MMP2, MMP9, and MMP12 of SCs. The degradation of matrix could not only provide tunnels or tracks for SC movement but also eliminate tissue barriers and form a TME for cancer metastasis.

## Conclusions

In summary, a reciprocal interaction between SCs and cervical cancer cells is revealed. SCs arrive at the site of cancer cells and secrete CCL2 as a strong chemoattractant which then induces CCR2^+^ cervical cancer cells to move along neurites. Conversely, cervical cancer cells upregulate the expression of MMPs in SCs to generate a suitable TME for SCs movement (Figure 7F). The CCL2/CCR2 axis might provide a prospective marker to predict PNI and affect NSRH indications.

## Data Availability Statement

All datasets generated for this study are included in the article/ Supplementary Material.

## Ethics Statement

The ethical approval for this study was obtained from the Institutional Ethics Committee of the International Peace Maternity and Child Health Hospital (IPMCH), number: [GKLW] 2017-125.

## Author Contributions

TH and QF designed the experiments and supervised the completion of this work. TH, YiW, and YC performed the experiments. TH and ZW analyzed and validated the results. TH wrote the original draft. XS, LY, YC, and YuW reviewed and edited the manuscript. XS and YuW were responsible for funding acquisition. All authors have read and approved the final manuscript.

### Conflict of Interest

The authors declare that the research was conducted in the absence of any commercial or financial relationships that could be construed as a potential conflict of interest.

## Supplementary Material

Supplementary Figure 1 Screening of cervical cancer cell lines prone to PNI. **(A)** Seven cervical cancer cell lines were co-cultured with DRG at day 2 (50× magnification). (**B)** The mean number of contact sites of seven cervical cancer cell lines after co-cultivation for 3 days. **(C)** Double immunofluorescence staining of neurites and ME-180 cells in the perineural niche. Staining: CM-DiI, NF-H, and overlay respectively (100× magnification, scale bar, 100 μm).

Supplementary Figure 2 DRG promotes the invasion of both cervical cancer cells and SCs arrived at the site of ME-180 cells. **(A)** DM promotes cell invasion of HeLa and ME-180 cells compared with CM. DM, DRG medium; CM, Control medium. **(B)** Cocultivation of DRGs with ME-180 cells. Images from confocal microscopy shows the process of SCs arrive at the sites of cancer cells, link to each other and induce ME-180 cells (marked by asterisks) moving toward DRG. Assume that the time of the first picture is 0 h. The SCs are marked by white arrows. The white dotted line describes the border of the Matrigel edge.

Supplementary Figure 3 Expression levels of MMPs in SCs and cervical cancer cells. **(A)** The fluorescent identification of rat Schwann cells with an S100β antibody (200× magnification, scale bar, 50 μm). **(B)** The concentration of MMP9 was significantly increased in the co-culture media. **(C)** MMPs expression in cervical cancer cells was not changed by co-cultivation with SCs. **(D)** Cervical cancer cells induced the upregulation of MMP2 and MMP9 *in vivo*. H&E-stained images are shown in the left (50× magnification, scale bar, 200 μm). MMP9- and MMP2-stained images are shown in the middle and right, respectively (200× magnification, scale bar, 50 μm). **(E)** Cancer cells adjacent to a peripheral nerve, staining from left to the right: overlay of PGP9.5/pan-CK/DAPI and Collagen IV/pan-CK/DAPI, showed lower expression of Collagen IV than cancer cells distant to nerves.

Supplementary Figure 4 The expression of CCL2/CCR2 and its effect on migration of cancer cells. **(A)** CCL2 concentration in the supernatant measured by ELISA (ns, not significant, and ^**^*P* < 0.01 compared to the DRG medium group). **(B)** Increased CCR2 expression in HeLa and ME-180 cells after co-cultivation with SCs. **(C,D)** Representative images of wound healing assays, 24 h after the scratch. The right image shows the statistical results. ^*^*P* < 0.05 (100× magnification, scale bar, 100 μm).

## Abbreviations

PNI: perineural invasion
SCs: Schwann cells
CCL2: Chemokine (C-C motif) ligand 2
CCR2: CCL2 receptor
EMT: epithelial-mesenchymal transition
TME: tumor microenvironment
ECM: extracellular matrix
Pan-CK: pan-cytokeratin
DRG: dorsal root ganglion
MMPs: metalloproteinases
MMP9: metalloproteinase 9
MMP2: metalloproteinase 2
MMP12: metalloproteinase12
NSRH: nerve-sparing radical hysterectomy
SCC: squamous cell carcinoma
AC: adenocarcinoma.
