# Supplementary figures and images for: Schwann Cell-Derived CCL2 Promotes the Perineural Invasion of Cervical Cancer
Source: Front Oncol. 2020 Jan 29;10:19. doi: 10.3389/fonc.2020.00019 (PMC7000531; doi:10.3389/fonc.2020.00019)

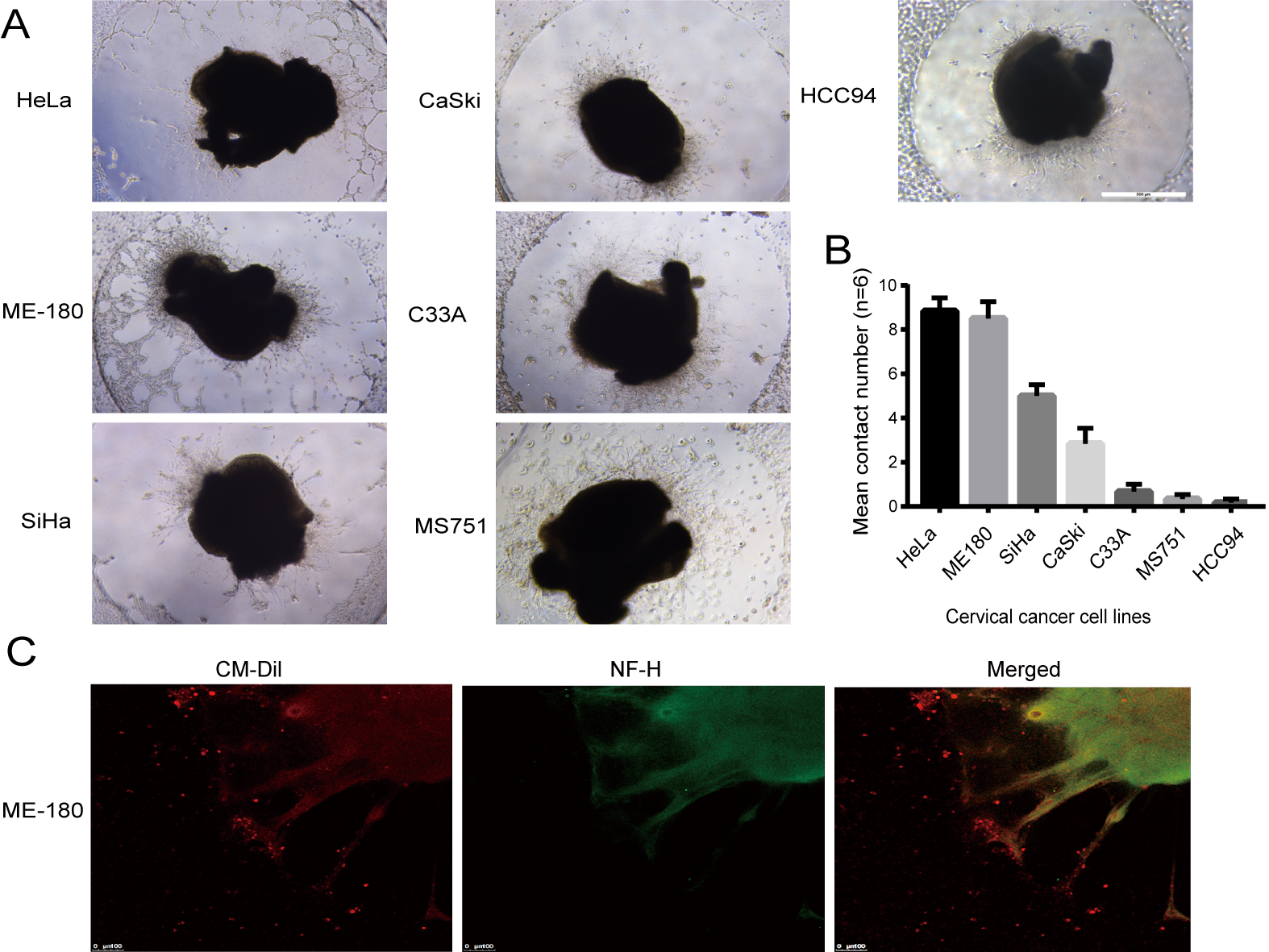

Supplement: Supplementary Figure 1 — Screening of cervical cancer cell lines prone to PNI. (A) Seven cervical cancer cell lines were co-cultured with DRG at day 2 (50× magnification). (B) The mean number of contact sites of seven cervical cancer cell lines after co-cultivation for 3 days. (C) Double immunofluorescence staining of neurites and ME-180 cells in the perineural niche. Staining: CM-DiI, NF-H, and overlay respectively (100× magnification, scale bar, 100 μm). [file Image_1.TIF]

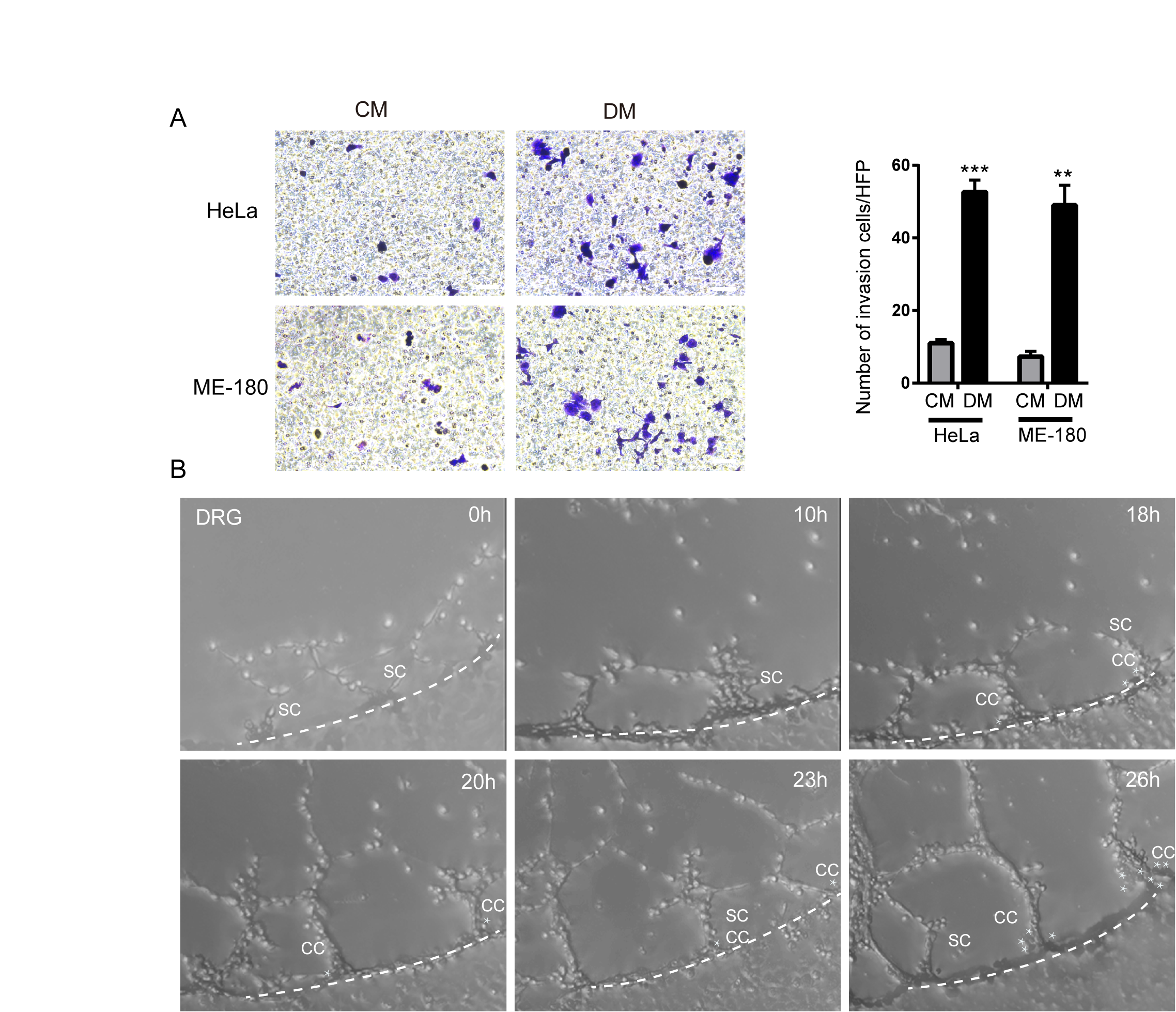

Supplement: Supplementary Figure 2 — DRG promotes the invasion of both cervical cancer cells and SCs arrived at the site of ME-180 cells. (A) DM promotes cell invasion of HeLa and ME-180 cells compared with CM. DM, DRG medium; CM, Control medium. (B) Cocultivation of DRGs with ME-180 cells. Images from confocal microscopy shows the process of SCs arrive at the sites of cancer cells, link to each other and induce ME-180 cells (marked by asterisks) moving toward DRG. Assume that the time of the first picture is 0 h. The SCs are marked by white arrows. The white dotted line describes the border of the Matrigel edge. [file Image_2.TIF]

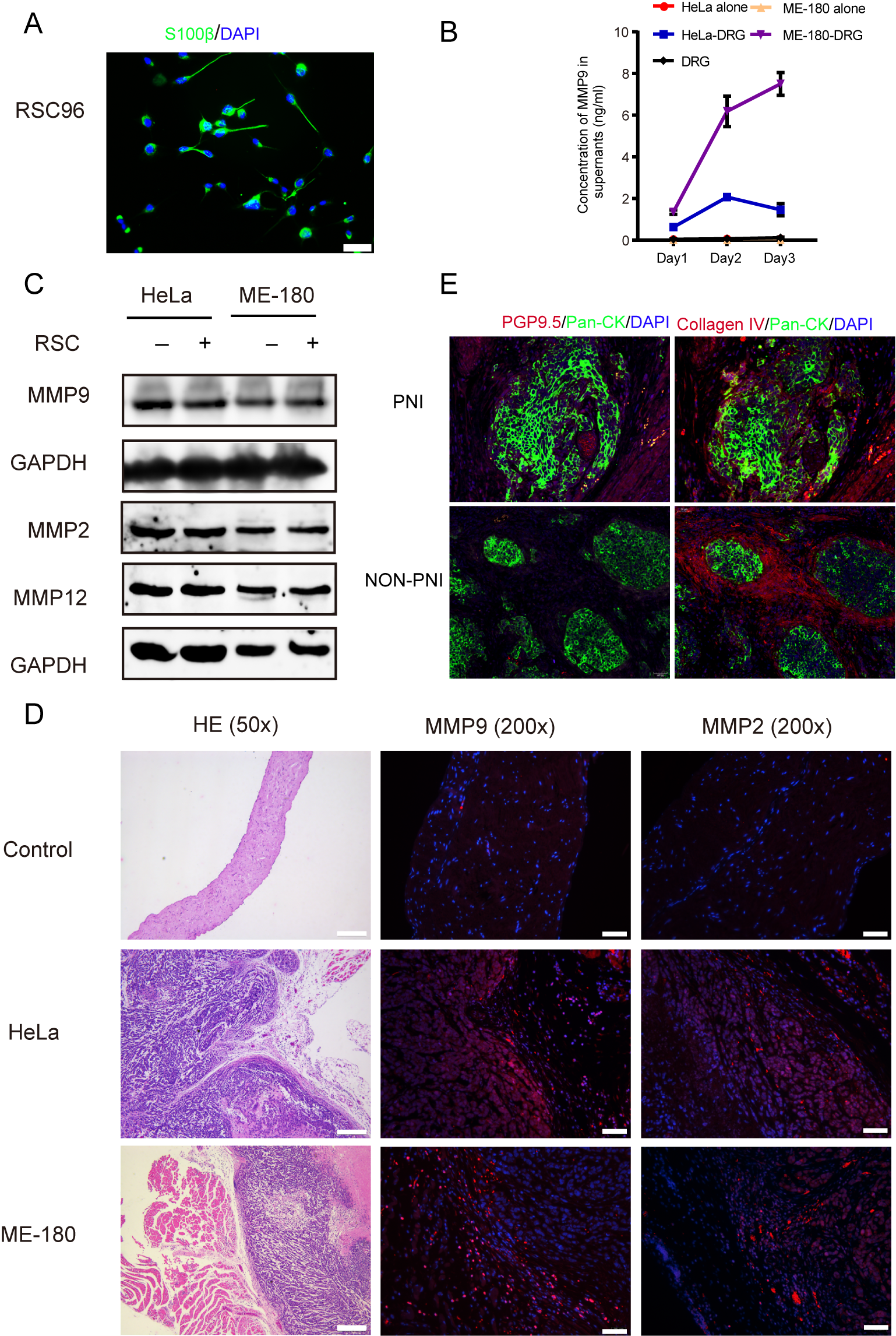

Supplement: Supplementary Figure 3 — Expression levels of MMPs in SCs and cervical cancer cells. (A) The fluorescent identification of rat Schwann cells with an S100β antibody (200× magnification, scale bar, 50 μm). (B) The concentration of MMP9 was significantly increased in the co-culture media. (C) MMPs expression in cervical cancer cells was not changed by co-cultivation with SCs. (D) Cervical cancer cells induced the upregulation of MMP2 and MMP9 in vivo. H&E-stained images are shown in the left (50× magnification, scale bar, 200 μm). MMP9- and MMP2-stained images are shown in the middle and right, respectively (200× magnification, scale bar, 50 μm). (E) Cancer cells adjacent to a peripheral nerve, staining from left to the right: overlay of PGP9.5/pan-CK/DAPI and Collagen IV/pan-CK/DAPI, showed lower expression of Collagen IV than cancer cells distant to nerves. [file Image_3.TIF]

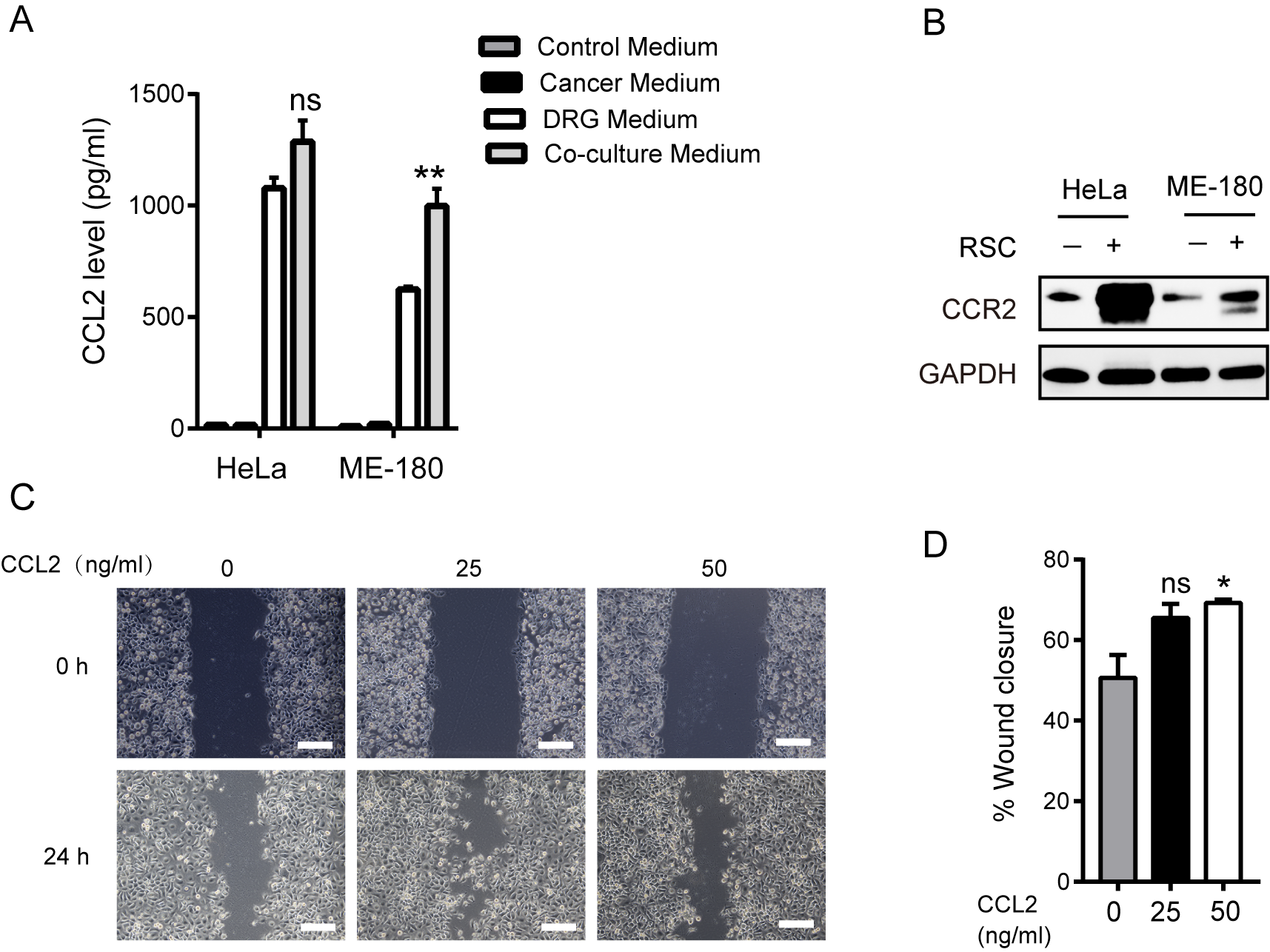

Supplement: Supplementary Figure 4 — The expression of CCL2/CCR2 and its effect on migration of cancer cells. (A) CCL2 concentration in the supernatant measured by ELISA (ns, not significant, and **P < 0.01 compared to the DRG medium group). (B) Increased CCR2 expression in HeLa and ME-180 cells after co-cultivation with SCs. (C,D) Representative images of wound healing assays, 24 h after the scratch. The right image shows the statistical results. *P < 0.05 (100× magnification, scale bar, 100 μm). [file Image_4.TIF]
